# Supplementary material for: Biomechanics of keratoconus: Two numerical studies
Source: PLoS One. 2023 Feb 2;18(2):e0278455. doi: 10.1371/journal.pone.0278455 (PMC9894483; doi:10.1371/journal.pone.0278455)
Supplement: S1 Fig — (a–d) Videokeratoscopy (a–b) and OCT (c–d) in a patient with diagnosed keratoconus in the left eye (a, c) and suspicion of subclinical keratoconus in the right eye (b, d). Videokeratoscopy showed posterior corneal elevation in the right eye whereas OCT showed no abnormal signs. (e–f) Videokeratoscopy in another patient with bilateral subclinical keratoconus. Visual acuity is preserved and the thickness of the corneas is almost normal but both the left (e) and right (f) eye exhibit posterior elevation. OCT, optical coherence tomography. (DOCX) [file pone.0278455.s001.docx]

# Supporting Information

# S1 Fig. Two cases of subclinical keratoconus.

(a–d) Videokeratoscopy (a, b) and OCT (c, d) in a patient with diagnosed keratoconus in the left eye (a, c) and suspicion of subclinical keratoconus in the right eye (b, d). Videokeratoscopy showed posterior corneal elevation in the right eye whereas OCT showed no abnormal signs. (e–f) Videokeratoscopy in another patient with bilateral subclinical keratoconus. Visual acuity is preserved and the thickness of the corneas is almost normal but both the left (e) and right (f) eye exhibit posterior elevation. OCT, optical coherence tomography.


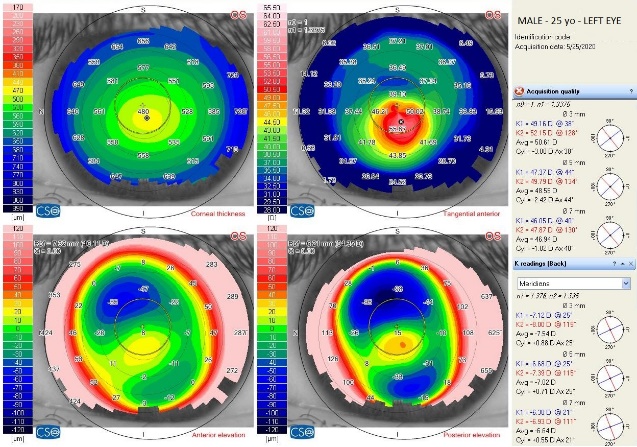


(a)


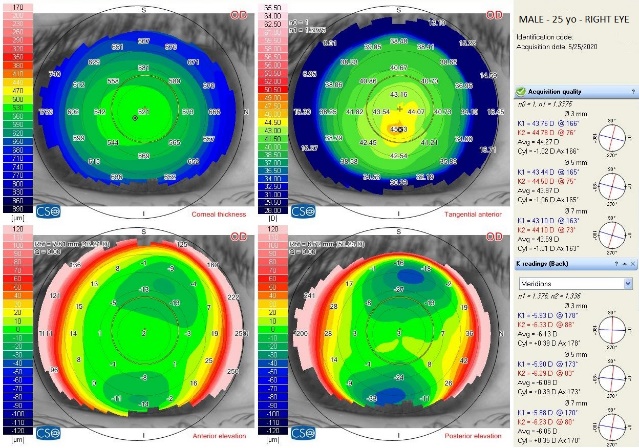


(b)

(c)


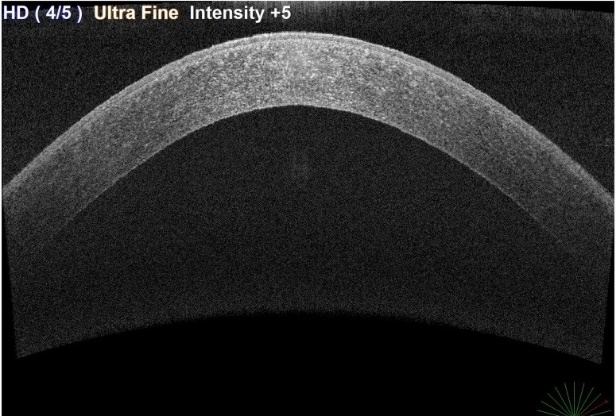


(d)


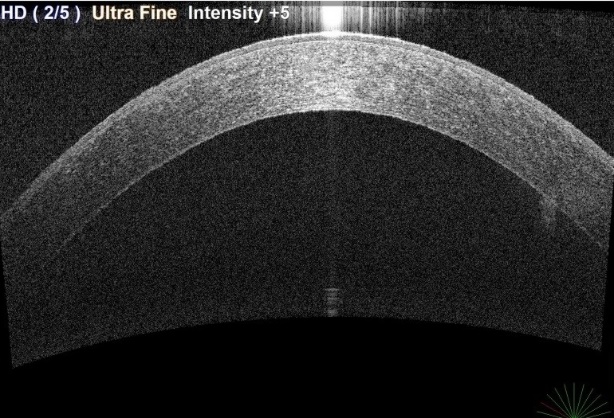

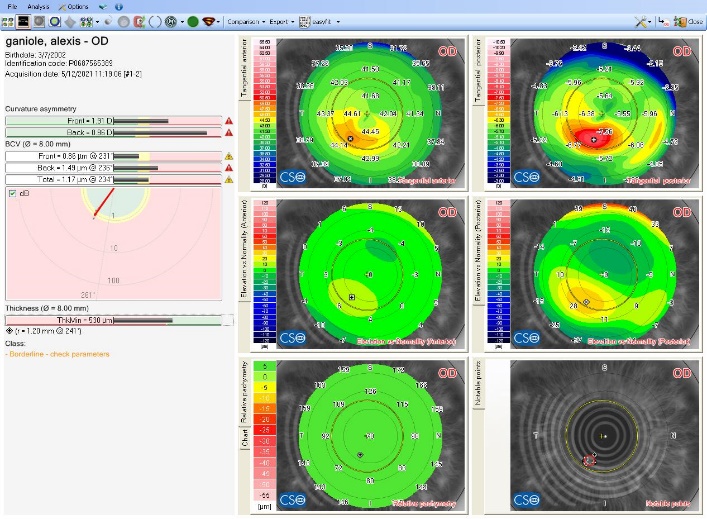

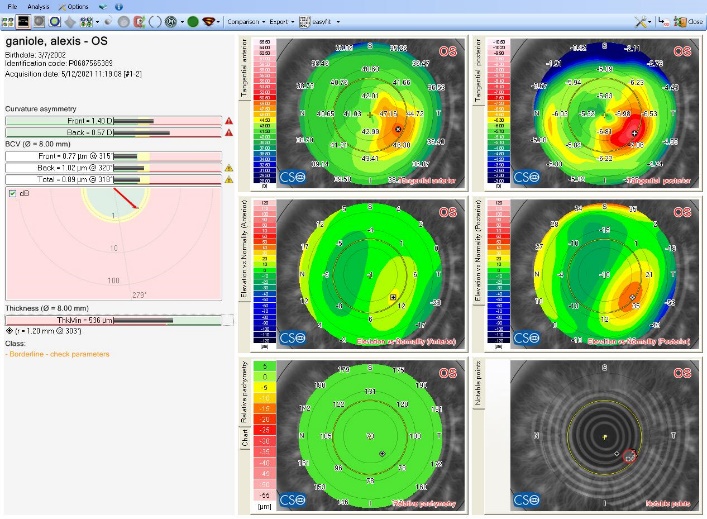


(e)

(f)
