# Supplementary figures and images for: Biomechanics of keratoconus: Two numerical studies
Source: PLoS One. 2023 Feb 2;18(2):e0278455. doi: 10.1371/journal.pone.0278455 (PMC9894483; doi:10.1371/journal.pone.0278455)

# Supporting Information

# S3 Fig. Mesh convergence study.


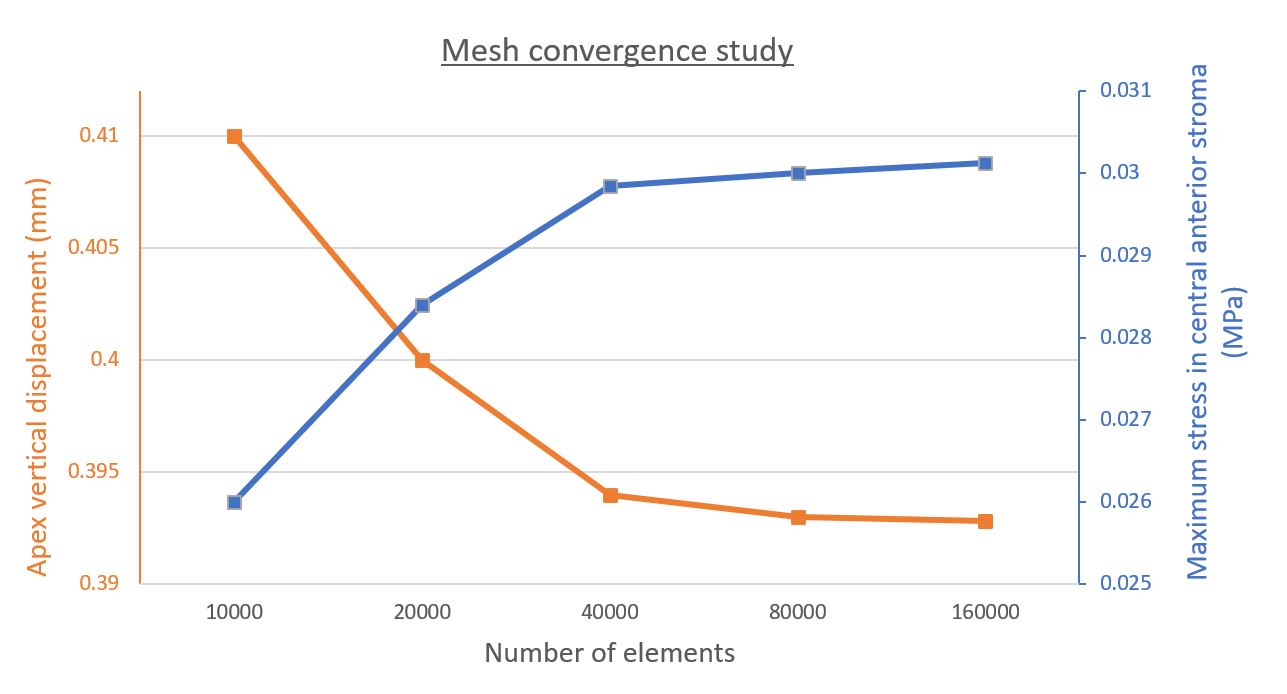

Supplement: S3 Fig — (DOCX) [file pone.0278455.s003.docx]
