# Supplementary material for: Biomechanics of keratoconus: Two numerical studies
Source: PLoS One. 2023 Feb 2;18(2):e0278455. doi: 10.1371/journal.pone.0278455 (PMC9894483; doi:10.1371/journal.pone.0278455)
Supplement: Attachment — Submitted filename: Response to reviewers (second).docx [file pone.0278455.s005.docx]

**Point-by-point responses to editor and reviewer comments (second)**

# **Responses to the editor**

Please, address the minor comments provided by Reviewer 2.

Reply: We have addressed the minor comments provided by Reviewer 2 by adapting Fig. 4 (we replaced the + signs with asterisks). The revised Figure was checked by PACE.

Journal requirements:

Please review your reference list to ensure that it is complete and correct. If you have cited papers that have been retracted, please include the rationale for doing so in the manuscript text, or remove these references and replace them with relevant current references. Any changes to the reference list should be mentioned in the rebuttal letter that accompanies your revised manuscript. If you need to cite a retracted article, indicate the article’s retracted status in the References list and also include a citation and full reference for the retraction notice.

Reply: Our reference list has been checked and is now correct: we removed one duplicate reference (refs 61 and 69 were the same) and corrected 11 refs. There were no retracted papers.

# **Responses to the Reviewers**

Reply: We thank the reviewers again for their time and for helping us improve our manuscript.

**Rev 2:**

'++' symbol has already been used for the "greatly increased softening" in the figure caption. So another symbol such as '+++' can be used here:
"++ indicates all layers were softened
961 to the same degree"

Reply: We decided to use two asterisks and have made the appropriate change to Figure 4 and the Figure 4 legend (Line 960).
